# Monitoring User Opinions and Side Effects on COVID-19 Vaccines in the Twittersphere: Infodemiology Study of Tweets

**DOI:** 10.2196/35115

**Published:** 2022-05-13

**Authors:** Beatrice Portelli, Simone Scaboro, Roberto Tonino, Emmanuele Chersoni, Enrico Santus, Giuseppe Serra

**Affiliations:** 1 Department of Mathematics, Computer Science and Physics University of Udine Udine Italy; 2 Department of Biology Università degli Studi di Napoli Federico II Napoli Italy; 3 Department of Chinese and Bilingual Studies The Hong Kong Polytechnic University Hung Hom Hong Kong; 4 Decision Science and Advanced Analytics for Medical Affairs and Pharmacovigilance Bayer Pharmaceuticals Whippany, NJ United States

**Keywords:** adverse drug events, COVID-19, digital pharmacovigilance, opinion mining, vaccines, social media, machine learning, deep learning, learning models, sentiment analysis, Twitter analysis, Twitter, web portal, public health

## Abstract

**Background:**

In the current phase of the COVID-19 pandemic, we are witnessing the most massive vaccine rollout in human history. Like any other drug, vaccines may cause unexpected side effects, which need to be investigated in a timely manner to minimize harm in the population. If not properly dealt with, side effects may also impact public trust in the vaccination campaigns carried out by national governments.

**Objective:**

Monitoring social media for the early identification of side effects, and understanding the public opinion on the vaccines are of paramount importance to ensure a successful and harmless rollout. The objective of this study was to create a web portal to monitor the opinion of social media users on COVID-19 vaccines, which can offer a tool for journalists, scientists, and users alike to visualize how the general public is reacting to the vaccination campaign.

**Methods:**

We developed a tool to analyze the public opinion on COVID-19 vaccines from Twitter, exploiting, among other techniques, a state-of-the-art system for the identification of adverse drug events on social media; natural language processing models for sentiment analysis; statistical tools; and open-source databases to visualize the trending hashtags, news articles, and their factuality. All modules of the system are displayed through an open web portal.

**Results:**

A set of 650,000 tweets was collected and analyzed in an ongoing process that was initiated in December 2020. The results of the analysis are made public on a web portal (updated daily), together with the processing tools and data. The data provide insights on public opinion about the vaccines and its change over time. For example, users show a high tendency to only share news from reliable sources when discussing COVID-19 vaccines (98% of the shared URLs). The general sentiment of Twitter users toward the vaccines is negative/neutral; however, the system is able to record fluctuations in the attitude toward specific vaccines in correspondence with specific events (eg, news about new outbreaks). The data also show how news coverage had a high impact on the set of discussed topics. To further investigate this point, we performed a more in-depth analysis of the data regarding the AstraZeneca vaccine. We observed how media coverage of blood clot–related side effects suddenly shifted the topic of public discussions regarding both the AstraZeneca and other vaccines. This became particularly evident when visualizing the most frequently discussed symptoms for the vaccines and comparing them month by month.

**Conclusions:**

We present a tool connected with a web portal to monitor and display some key aspects of the public’s reaction to COVID-19 vaccines. The system also provides an overview of the opinions of the Twittersphere through graphic representations, offering a tool for the extraction of suspected adverse events from tweets with a deep learning model.

## Introduction

### Background

The COVID-19 pandemic has been at the heart of the discussions on all media outlets for almost 2 years. These debates touch upon very important and sensitive topics such as health, politics, work, school, and personal freedom to cite only a few. In a general effort to tackle the pandemic, many countries have engaged in the fastest and most massive vaccine rollout witnessed in human history: in less than 1 year, several vaccines have been created, tested, and distributed around the world, and many others are at the last phase of clinical trials and/or waiting for approval from regulatory agencies [1]. Despite the great efforts put into development, the rollout of vaccines has been slowed down in various countries [2] due to hesitancy and fake news poisoning social media debates. The vaccination rollout for the first strains of the virus has proceeded slower than initially planned, and experts agree that it is imperative to find ways to accelerate future iterations to keep pace with the new COVID-19 variants [3]. One of the ways to improve this process is to study how the population reacted to the first vaccination campaigns, the types of information/misinformation shared, and the impact this had on vaccination hesitancy.

Social media platforms are, of course, one of the main stages of this debate.

In the last years, microblogging services such as Twitter have seen an increase in popularity due to their immediacy and ease of use. Moreover, brands, institutional bodies, politicians, public figures, and traditional news outlets have realized the importance of having a presence on these platforms, which allow them to deliver messages with high impact and unprecedented reach [4,5].

The rapid spread of the pandemic, fast development of the vaccines, and increasing worries about their safety have been hot topics on social media since the very beginning.

The vaccination campaigns planned by national governments could therefore be seriously hampered by misinformation on such outlets [6,7]. Many recent studies [8] have taken great interest in analyzing different social media platforms to track the sentiment of users about COVID-19 vaccinations across different cities [9], looking for the main misconceptions and complaints about the COVID-19 control measures [10] and the confidence in the efficacy of the vaccines [11].

These are only few examples demonstrating why monitoring social media platforms is a highly informative and beneficial approach to discover health-related issues (eg, detecting mentions of adverse events [AEs]) and to better understand public opinion (eg, monitoring the information quality and contrasting the spread of fake news). From this point of view, modern systems for digital pharmacovigilance can deploy natural language processing techniques to collect and analyze online discussions. This allows for the identification of potential AEs that may not have been detected during clinical trials, enabling timely decisions to reduce their harm. In the near future, it is likely that even public health care systems will increase their monitoring activities on social media platforms, with the goal of identifying and treating health issues such as mental diseases, managing information by contrasting fake news, or launching prevention campaigns (eg, to mitigate vaccine hesitancy) [12].

### Objective

**Figure 1:**
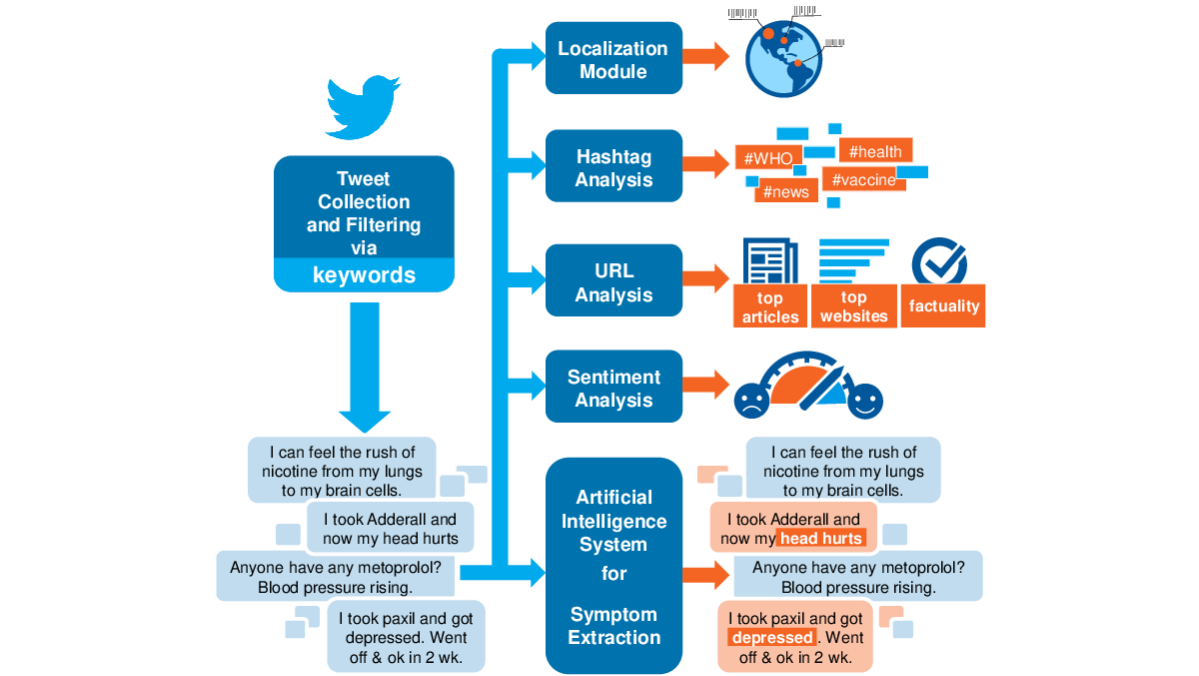
Schema of the full system architecture used to analyze the information displayed on the web portal.

We here present an overview of our system for monitoring and analyzing vaccine opinions. Its modules aim at generating insights from Twitter on the topic of COVID-19 vaccines. The tool collects tweets daily and analyzes them to extrapolate information about public reception of the vaccination campaigns on social media. The information on our interactive web portal is also broken down into easy-to-read charts for both specialized and general audiences. Figure 1 illustrates the architecture of the full system behind the web portal. The portal consists of a module dedicated to data collection and various modules dedicated to data processing. The main features of the system are: (1) Localization, (2) Hashtag Analysis, (3) News Sources Analysis, (4) Sentiment Analysis, and (5) Symptom Extraction.

The Symptom Extraction module, in particular, consists of a deep-learning architecture that we created specifically for this task, based on SpanBERT [13], an extension of the bidirectional encoder representations from transformers (BERT) model, which is one of the state-of-the-art models for AE detection [14-16].

Each processing module is built to extract specific information from the collected tweets (eg, the most used hashtag or the most shared links). This information is then cleaned and provided to the user through the web portal with interactive charts and diagrams. To ensure greater readability, colors and shapes were preferred over figures when presenting the data.

To summarize, our objective was to present a tool for the collection and processing of data on COVID-19 vaccines, followed by their visualization on a web dashboard [17].

In contrast to related previous works, we focused on monitoring tweets about specific vaccines. This allowed us to compare their public reception and how it changes over time. Besides combining various features that can be found separately in recent works, we also introduced innovative modules (eg, Symptom Extraction), which can offer new insights on the related public discourse.

The code for the data collection and the preprocessing tools, as well as all the precomputed statistics and the IDs of the tweets, can be openly accessed from GitHub [18]. The amount and type of data that can be shared openly are limited by Twitter’s privacy policy. However, further information can be requested for research purposes. We also present a case study on the AstraZeneca vaccine, as an example of the analyses that can be carried out on the data using our system.

### Related Work

Since the start of the COVID-19 pandemic, organizations worldwide have stressed the need to collect and share all data available on the virus, its effects, and all related research [19]. As time passed, these resources grew in size, and some researchers also started analyzing data coming from social media.

For example, Kwok et al [10] collected 31,100 Australian tweets (from January 20, 2020, to October 22, 2020) related to COVID-19 vaccines. Their paper focuses on analyzing the sentiment and opinion of the users about the vaccines and the main recurring topics in the tweets. Similarly, Yan et al [9] collected and analyzed Reddit comments about COVID-19 vaccines from three Canadian cities (from July 13, 2020, to June 14, 2021), and performed a comparison of the sentiment and main discussion topics among the three locations. Other recent works focused on analyzing sentiment and discussion topics in tweets about COVID-19 generated in other countries and in different time periods [20-22].

These works were carried out on very specific time periods, which focused on a single aspect of the social media messages. A more comprehensive study was carried out on AvaxTweets [23], a public data set of Twitter posts and accounts that exhibited a strong stance against COVID-19 vaccines, collected between October 2020 and December 2020. The authors analyzed the accounts in terms of the most frequent hashtags, which news sources they shared, and their most likely political orientation, looking for useful insights on how to counter misinformation and vaccine hesitancy. However, both this and the preceding works were carried out on a limited time scale and aimed specifically at the research community, providing no tools or web interfaces to explore the data.

At the same time, various researchers focused not only on data collection but also on ways to start processing and visualizing the data to make them available for a broader public. COnVIDa [24] is a web-based platform that provides day-to-day interactive information on COVID-19–related conditions in Spain, collating data from various sources (eg, health databases, mortality reports, statistics, information on citizens’ mobility from Google and Apple Maps). This project focuses on a single country and tries to combine different aspects of the situation to give the viewer a more complete visualization. CoVaxxy [25] is another data set and online dashboard that focuses on the correlations between tweets about COVID-19 vaccines, credibility of the shared news, and vaccine adoption on US geolocated posts. Sharma et al [26] presented another recent tool, which was used to collect and analyze Twitter conversations from March 1, 2020, to June 5, 2020. The dashboard visualizes sentiment information and trending topics, but focuses particularly on the credibility of the news shared in the tweets and on how misinformation spreads.

Our proposed system includes many of the features offered by these previous works, such as continuous day-to-day data collection and processing (since December 15, 2020), global data collection (not country-specific), sentiment analysis, and news sources analysis. Our tool differs from these previous works in relation to the following aspects: (1) focused monitoring of specific vaccines since the date of their approval, which enables users to compare the public’s reaction to them; (2) a wide variety of processing modules (not focused on a single aspect) to provide a multifaced view of the social media discourse; (3) a comprehensive dashboard to visualize all of the processed data in an easy-to-read manner for different categories of users; (4) an innovative symptom extraction module to track the most discussed side effects; and (5) openly available code and data.

## Methods

### Data Collection

Tweets are collected using the Twitter application programming interface (API) [27]. To recover the most recent tweets mentioning a specific vaccine, we use the query “covid vaccine <vaccine_name>,” where <vaccine_name> is the lowercase name of one of the monitored vaccines (originally Pfizer-BioNTech, AstraZeneca, and Moderna, which was then expanded to include the newly introduced vaccines). We require that all keywords are present in the tweet (either as text, hashtag, or as part of a link in the tweet) and that each query contains the name of only one vaccine.

Tweets are selected among the “most recent,” as opposed to the “most popular,” and retweets are discarded. This is done to avoid skewing the data with popular tweets produced by few influential users. Although we are collecting tweets in various languages, only those written in English are passed to the following stages of processing, as most of our current modules are language-dependent. Nonetheless, we are storing these data for future research, as we plan to overcome this limitation in the near future with the introduction of multilingual models (in particular for AE detection and sentiment analysis) and automated translation services. This will allow us to perform a complete analysis for all monitored languages.

The query is run every 24 hours, with a cap of 7000 requested tweets per day (to be divided among the monitored vaccines) imposed by the limits of the API. Despite the theoretical limitation, the number of new tweets that matched the query in the last 24 hours never exceeded 7000.

The body of the remaining messages undergoes additional preprocessing steps to identify possible duplicates and discard tweets that are practically identical (apart from hashtags, punctuation, or URLs). This situation occurs, for example, when users share a piece of news using the “Share on Twitter” button provided by news websites. If the user simply shares the news without adding any comments (or adding only a hashtag), the result is a high number of nearly identical tweets that do not provide additional information aside from the fact that the particular piece of news was shared multiple times. Such tweets are marked as “duplicated,” but are not discarded because they can provide useful information on which articles went viral; nevertheless, they are marked to avoid introducing noise into other types of analyses.

Deduplication is performed by removing all hashtags, URLs, and punctuation, followed by (fuzzy) matching with the collection of “unique” tweets already collected.

Data collection started on December 10, 2020, concurrent with the Food and Drug Administration approval of the first COVID-19 vaccine (Pfizer-BioNTech), and the system has currently (September 7, 2021) analyzed over 650,000 tweets. Table 1 presents the names of the vaccines tracked at the time of writing and the date we started collecting related data.

**Table 1:** Names of the tracked vaccines and dates on which data collection started.

Vaccine name	Start date
Pfizer-BioNTech	December 10, 2020
AstraZeneca	December 11, 2020
Moderna	December 16, 2020
Sinopharm	February 24, 2021
Sputnik V	February 24, 2021
Sinovac	February 24, 2021
Johnson & Johnson	April 1, 2021

### Ethics Considerations

Twitter is a major social network and, as such, has strict policies to regulate the ethical use of its data and the privacy of its users. Following their guidelines, we collect and store only the information needed for the processing steps that are currently implemented. We memorize the outputs of the modules and discard all of the sensitive data soon afterward. We also memorize the tweet ID, which allows us (and other researchers) to access the original tweet in the future, as long as the user does not delete it or change its visibility.

If a tweet needs to be displayed on a web interface, we use the API provided by Twitter, which allows us to display tweets on demand given their tweet ID (and only if their current visibility settings allow them to be displayed).

### Data Processing of Incoming Data

#### Localization Module

The localization module enables tracking the geographical origin of the tweet, visualizing which countries are more involved in the discussion about the vaccines.

The geolocation is extracted directly from the tweet whenever possible. Users on Twitter can decide whether to share their location or not at any moment, and whether to geotag the places mentioned in their tweets. If the precise geolocation is not available, the module attempts to reconstruct it using the user’s “location,” a free-text field located in the user’s profile. As such, “location” may contain imaginative terms or nonexistent locations (eg, “over the rainbow” or “the universe”). The module relies on heavy preprocessing, normalization, and cleaning steps to discard most of the noisy locations. The remaining locations are passed on to Google Maps services [28] to determine the most accurate match.

The information is displayed on the web portal as a world map, where countries are shown in different shades of color; the larger the number of tweets coming from that country, the darker the color (the scale is exponential).

#### Hashtag Analysis

Hashtags are extracted from the most recent tweets only (the last 7 days, updated daily). We automatically remove a curated selection of hashtags, considered to be of low information content. In particular, we remove all hashtags containing the name of the vaccines that we are tracking (eg, #pfizer, #moderna, #biontech), words directly related to COVID-19 (eg, #covid, #coronavirus, #covidvaccine), and those containing the term “vaccine” only.

Information displayed on our web portal shows the hashtags as a colored treemap, where most of the tweeted hashtags cover a wider area and are darker in color.

#### News Sources Analysis

Sensitive topics such as health and vaccinations are fertile ground for the spread of misinformation, as proven by the amount of COVID-19–related fake news, which have been debunked in 2020 by fact-checking agencies (eg, PolitiFact [29]) and the precautions taken by the major social networks when dealing with posts mentioning the pandemic (eg, Facebook [30]).

An analysis of the most shared articles is of key importance to understand which sources of information are used by the public to inquire about vaccines.

We run the analysis by collecting all URLs contained in the tweets. We consider the most recent tweets only (last 7 days, updated daily) to reflect the impact of the most recent news. URLs are used both in their full form and considering their domain only. Unique URLs and domains are counted and used to provide two different kinds of information: the single most shared webpages (to individuate trending articles) and the most popular sources of information (intended as websites/domains, to individuate the favorite source of information in general).

#### Factuality Analysis

To further investigate the factuality of the URLs shared by users, we make use of Iffy+ [31], a website that provides an updated list of websites ranked by their factuality level. The lists provided by Iffy are the result of an aggregation of different popular fact-checking websites and trusted sources (eg, FactCheck.org, PolitiFact, and Wikipedia). The list we take into account is composed, for the most part, of websites with a low Media Bias/Fact Check (MBFC) factual level [32] and sources of fake news/misinformation identified by BuzzFeed, FactCheck.org, PolitiFact, and Wikipedia. We use this list to perform a factuality analysis over all of the collected tweets.

For each URL in a tweet, we check if its domain belongs to one of the websites on the Iffy+ list. If it does, we classify it according to its level of *MBFC factuality* (high, mixed, low, very low), and its *misinformation category* (eg, conspiracy, fake news). Factuality level and misinformation category might be not available for some of the websites (“not available”). If a domain is not part of the Iffy+ list, we assume it is a reliable (“reliable”) source of information. All domains with a factuality level greater than or equal to “high” are labeled as “reliable.” Only 0.0089% of the “reliable” URLs fall into this category.

We want to highlight that this analysis only explores the reliability of the links that the users are sharing, but not the legitimacy of the tweet as a whole. For example, a user might share a “fake news” article as a way to joke, mocking it in the text of the tweet. There might also be cases of users sharing links from reliable sources, accompanied by inflammatory or fake captions.

#### Sentiment Analysis

The sentiment analysis module aims at understanding the attitude of the users when sharing their opinions of the vaccines and their possible side effects. To understand the general sentiment of the crowd when talking about the vaccines, we employ a RoBERTa model [33] trained on tweets, which was fine-tuned for the sentiment analysis on the TweetEval Benchmark [34,35]. The model reached a macroaveraged recall of 72.6 (SD 0.4) on the test set.

This type of module is useful to interpret the general mood of the people speaking about the vaccines, about their possible side effects, or even about their vaccination experiences. In particular, this can be very effective to understand if a user is reporting facts, expressing distress, or expressing a positive attitude. For each tweet, the sentiment calculated using RoBERTa is normalized to a discrete set of values (positive, negative, or neutral) for ease of visualization.

Our web portal features an interactive line graph to observe how the sentiment varies in time. It allows the visitor to inspect the sentiment globally and compare the trends for the tweets mentioning specific vaccines.

#### Symptom Extraction

In the last decade, people have started discussing their personal health status on social media more and more often, looking for users with similar experiences, asking for suggestions, or reporting unexpected effects after the assumption of medicines. The latter represents an interesting type of information, as these effects might be considered as AE indicators for pharmacovigilance purposes.

Systems for the automatic extraction of AEs from informal and social media texts are at the core of a growing research trend in the field of natural language processing [36,37]. Moreover, several shared tasks have been recently organized within the audit command language community [38,39] to raise interest about this topic.

We evaluated different combinations of transformer-pretrained models and conditional random fields (CRFs) to create an effective deep-learning architecture for the task [16]. The best-performing model employs a neural network architecture based on SpanBERT [13] and CRFs [40], trained on the Adverse Event Detection data set of the Fourth Social Media Mining for Health Applications Shared Task (SMM4H) [41], thus representing the current state of the art on the Shared Task [14,15] (Table 2).

These evaluation metrics resemble more closely how humans might perceive the correctness of the predictions. The AE extraction problem is modeled as token classification, tagging each word in the text as “inside” or “outside” of a symptom/AE.

The samples go through five main processing steps: text preprocessing, subword tokenization, BERT modeling, intermediate label prediction, CRF, final label aggregation.

**Table 2:** Performance of our adverse event extraction module against the previous top-performing models on the Fourth Social Media Mining for Health Applications Shared Task 2019.^a^

Architecture	Relaxed metrics^b^			Strict metrics		
	F1	Precision	Recall	F1	Precision	Recall
SpanBERT^c^+CRF^d^ [15]	70.2	60.8	83.0	46.4	39.6	56.1
KFU [42]	65.8	55.4	81.0	46.4	38.9	57.9
THU_NGN [43]	65.3	61.4	69.7	35.6	32.8	38.8
MIDAS@IIITD [44]	64.1	53.7	79.3	32.8	27.4	40.9
TMRLeiden [45]	62.5	55.5	71.5	43.1	38.1	49.5

^a^Data were obtained from the public CodaLab leaderboard [46].

^b^Relaxed evaluation of the model’s performances. A prediction that does not match exactly the correct adverse event, but overlaps with it (eg, “headache” instead of “strong headache”) is not discarded but considered as a “partial match” (worth half a point).

^c^BERT: bidirectional encoder representations from transformers.

^d^CRF: conditional random field.

The module of our system extracts all symptoms that are being discussed in the tweets. The data are then aggregated and visualized on the web portal as a word cloud. The data can be filtered by vaccine and by period of time to discover what concepts the crowd focused on at different stages of the vaccination campaign.

**Figure 2:**
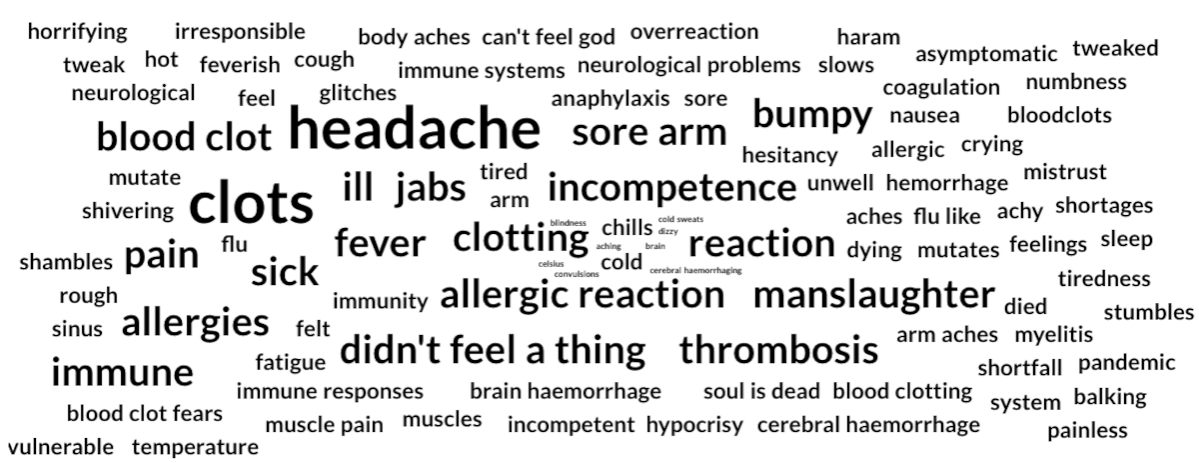
Possible side effects of the AstraZeneca vaccine, as discussed on Twitter. The word cloud was generated using our adverse event extraction model and displayed on the web portal. The size of the words is proportional to their frequency.

Figure 2 shows an example of the word cloud generated using tweets regarding the AstraZeneca vaccine following the thromboembolic events reported in several European countries during March 2021 [47].

### Model Validation

The Sentiment Analysis and Symptom Extraction modules are based on deep-learning models, and it is thus crucial to verify their generalization capabilities outside benchmark environments. To more rigorously evaluate the performance of the modules mentioned above, we sampled and annotated a subset of the collected tweets to compare the model’s predictions with human ground-truth labels on real-world data.

A total of 1000 tweets were extracted using stratified sampling to maintain the same distribution of tweets over months. Three annotators with high English proficiency (C1) were tasked to mark the sentiment of the tweets on a three-point scale (positive, neutral, negative) and highlight any vaccine-related AEs mentioned in them.

The gold sentiment of the tweet was decided by majority vote. The gold adverse events of the tweets were decided as the set of all sequences of words that were highlighted by at least 2 out of 3 annotators. For example, if the annotations were “strong headache,” “headache,” and “having a strong headache,” the final annotation would be “headache.”

The human-generated annotations were used as ground truth to evaluate the performance of the two deep-learning modules on the real-world data and compare them with their performance on the benchmark data sets.

## Results

### Overall Results

First, we performed an initial analysis on the number of unique tweets and unique user accounts present in the collected data. As mentioned in the *Data Collection* subsection of the Methods, we took some precautions to avoid collecting duplicated data or skewing the data set by giving more weight to tweets posted by popular accounts. To verify if these strategies were successful, we inspected the ratio of unique tweets and users in the data set, month by month and overall.

**Figure 3:**
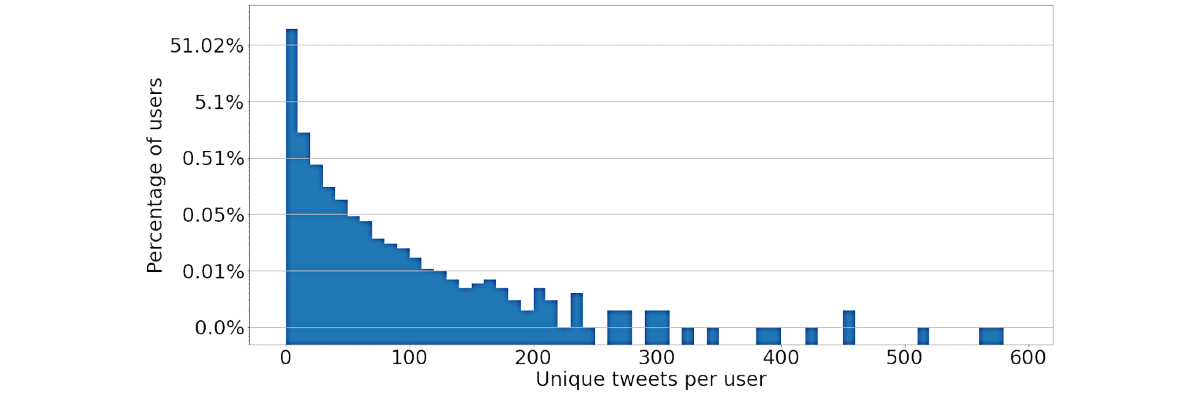
Distribution of users depending on how many times they tweeted (the y axis is presented in logarithmic scale).

Figure 3 shows the distribution of users depending on how many times their tweets appeared in the data set. We can clearly see a long-tail distribution, where 75% of the users only tweeted once, 92% of users tweeted at most three times, and 98% of users tweeted at most 10 times (ie, on average once per month). Looking at the users that tweeted more, most of them were news outlets, who tweeted from 50 to 578 times in the considered timespan (0.18% of the total users). The long-tail distribution is a good sign, as it shows that most of the users from whom we collected tweets are likely regular users and not influencers or content farms.

**Figure 4:**
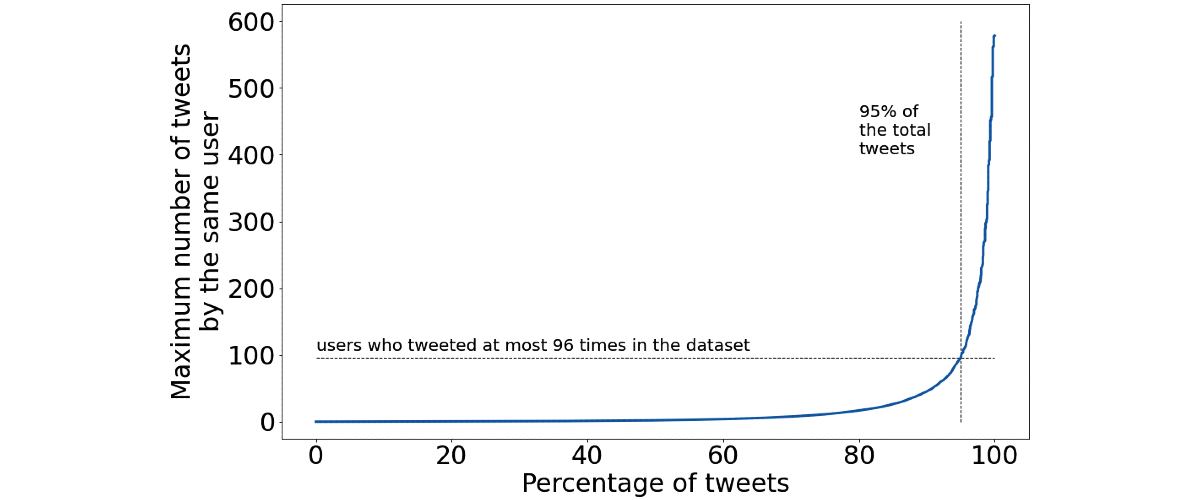
Percentage of tweets produced by a group of users, depending on how many tweets the user produced; 95% of the tweets in the data set are produced by users who tweeted at most 96 times in the considered timespan.

We then looked at the origin of the tweets that composed the data set. Figure 4 shows that 95% of the total tweets were posted by users that tweeted less than 100 times in the considered timeframe. This is another positive indication that the collection of tweets is not heavily influenced by a small number of super accounts, and thus the subsequent analysis should not suffer from this kind of bias.

Finally, we calculated some statistics on a monthly basis, which are reported in Table 3. The mode and median were 1, confirming the findings discussed above. The average number of tweets per user remained stable at around 1.4 during the first months (December 2020 to March 2021). This number then increased to 1.5 in the period between April and June, following the start of the vaccination campaigns and the AstraZeneca controversy (likely due to heightened news coverage). Following June, the average number of tweets per user went down again.

The number of unique tweets and unique users considered each month was roughly stable.

**Table 3:** Statistics on the unique number of tweets and users for each month in the collected data set.

Month	Unique tweets, n	Unique users, n	Tweets per user			
			Maximum	Mean (SD)	Mode	Median
December 2020^a^	21,235	15,983	40	1.32 (1.29)	1	1
January 2021	42,891	30,294	71	1.42 (1.76)	1	1
February 2021	36,897	25,102	98	1.47 (1.98)	1	1
March 2021	51,469	35,402	181	1.45 (2.47)	1	1
April 2021	62,697	41,160	117	1.52 (2.45)	1	1
May 2021	48,785	32,263	134	1.51 (2.45)	1	1
June 2021	41,364	27,397	154	1.51 (2.45)	1	1
July 2021	42,742	29,371	139	1.46 (2.26)	1	1
August 2021	41,596	29,942	232	1.39 (2.09)	1	1
September 2021^a^	7064	5833	27	1.21 (0.84)	1	1
All	396,740	196011	578	2.02 (6.19)	1	1

^a^Partial data, not spanning the entirety of the month.

### Localization

Since we are only considering English-language tweets, the most active countries were the United States, Canada, and the United Kingdom; followed by Nigeria, India, and Australia; and finally various European countries. Despite the language limitation that we imposed, the system detected tweets from almost all countries in the world.

We plan to remove the language limitation in the near future by means of the usage of automated translation services.

### Hashtags

Most of the top hashtags were related to the concepts of “health,” “news,” or mentioned specific countries that made it to the top headlines due to recent outbreaks and similar accidents.

### News Sources

The current data show a reassuring trend: the most popular sources of information are renowned newspapers (such as The New York Times or The Guardian), official institutional websites (eg, www.gov.uk), and scientific authorities (eg, the European Medicines Agency [EMA] and World Health Organization). It is also interesting to note that since the monitoring started in December 2020, the video-sharing platform YouTube has always been among the top-15 most shared domains. The top-5 most shared articles are displayed on the website as clickable links (displaying the URL and title of the page), while the 15 most popular domains are shown as a bar graph.

### Factuality

**Figure 5:**
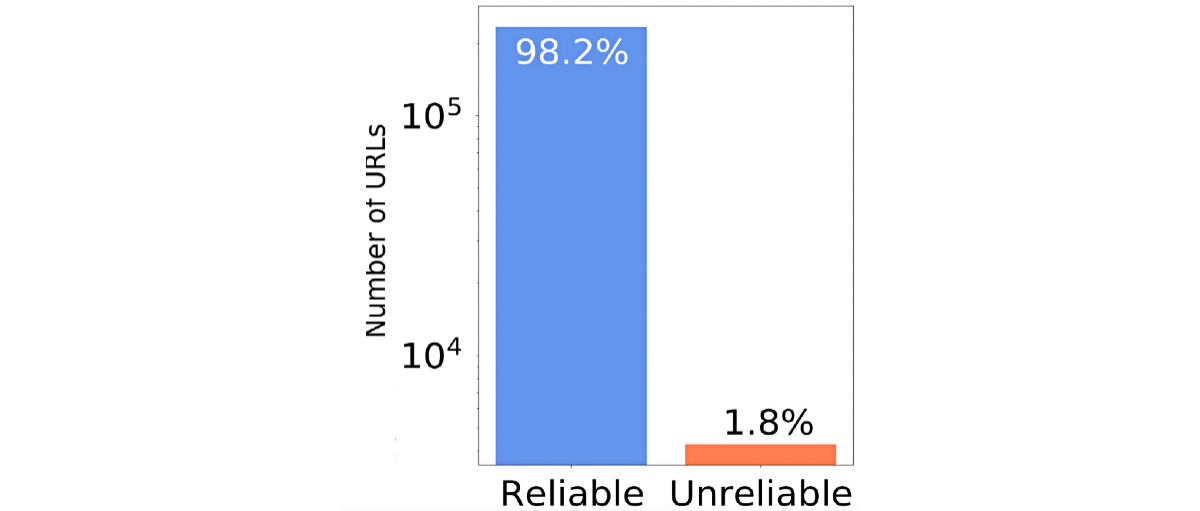
Percentage of the Reliable and Unreliable URLs shared (y axis is presented in logarithmic scale).

The vast majority of the shared URLs were classified as having a “reliable” level of factuality (98%, see Figure 5). This seems to be confirmed if we look at the five most shared domains: theguardian.com (3.22%), nytimes.com (2.75%), reuters.com (2.40%), cnbc.com (1.77%), and abc.net.au (1.56%).

**Figure 6:**
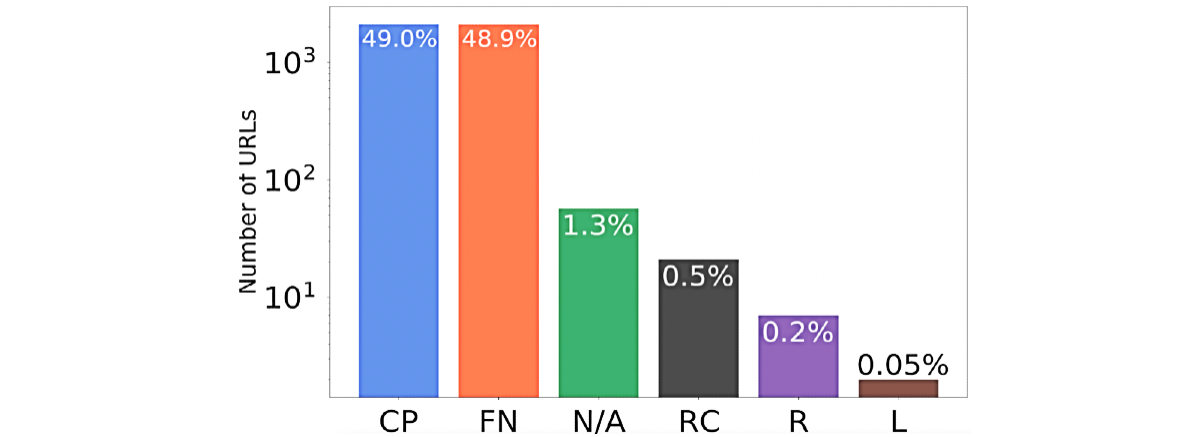
Distribution of Media Bias/Fact Check misinformation categories for “Unreliable” URLs. The y axis is presented in logarithmic scale. CP: Conspiracy-Pseudoscience; FN: Fake-News; N/A: Not Available; RC: Right-Center bias; R: Right bias; L: Left bias.

The remaining 2% was composed of domains classified mostly as *low* and *mixed* (ie, a website that is known to share both factual and nonfactual information). Figure 6 shows the factuality distribution of “unreliable” URLs (note that these data are presented on the logarithmic scale).

**Figure 7:**
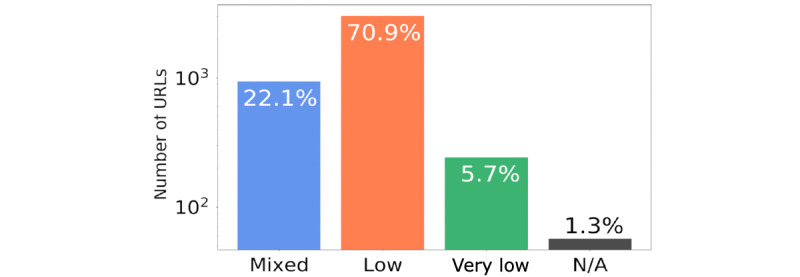
Distribution of Media Bias/Fact Check factuality level for “Unreliable” URLs. The y axis is presented in logarithmic scale. N/A: not applicable.

Looking at the misinformation categories for the “unreliable” domains (Figure 7), 49% were classified as “Conspiracy-Pseudoscience,” 49% as generic “Fake-News” sources, and the remaining were subject to political biases.

### Sentiment Analysis

The global sentiment of the analyzed tweets was neutral/negative for most of the period of observation, with occasional spikes of positivity for individual vaccines. The negative trend might be enhanced by the fact that shocking, controversial, or tragic news tend to be shared and spread more easily on the internet when compared with other kinds of news.

### Symptom Extraction

In the days preceding March 11, the most prominent concepts in AstraZeneca’s word cloud were “headache” and “fever”; however, as soon as thromboembolic events started being discussed on the internet, the system detected the shift in topic, and words such as “clots” and “thrombosis” quickly became noticeable in the cloud.

With regard to the other two vaccines, “allergic reactions,” “headache,” and “fever” were consistently among the most shared and discussed AEs. “Anaphylaxis” was one of the major concepts on Pfizer-BioNTech’s cloud for a long period of time at the beginning of the vaccination campaign, but is now slowly losing traction (this is evident in the word cloud on our web portal [17]).

This model could identify tweets containing potential AEs and highlight the mention of the symptoms. However, there are no mechanisms in place to verify the reliability of the tweets and there is no human fact-checking involved in the process. This means that, for the time being, there is virtually no distinction between symptoms that were actually reported by the users and exaggerations or hoaxes. This limitation is clearly stated on the web portal and the viewers are encouraged to further inspect the tweets on their own to have a clearer idea of what kind of messages lead to the prediction of the extracted symptoms. Clicking on any word in the word cloud displays a selection of the analyzed tweets that mentioned that concept in the selected time period.

The section “Evolution of mentioned symptoms over time” contains an analysis of the information that can be extracted by the representations produced by this module.

Finally, we would like to recall that the system was trained solely on the data provided during the SMM4H 2019 Shared Task. Even though it is one of the best performing models on this task, the model still suffers from the limitations of current AE extraction systems, such as the difficulty in making reliable distinctions between side effects (caused by medications), symptoms (caused by illnesses), and the names or descriptions of some medical conditions. For example, in the sentence “I have a *slipped vertebrae* and a *degenerative disk*,” the two medical conditions are identified as side effects by the system.

This is a common problem for such systems, which are often trained on data sets that are limited in size and linguistic variety.

### Model Validation

We experimentally evaluated the performance of both the Sentiment and Symptom Extraction modules using the subset of 1000 manually annotated tweets we created.

**Figure 8:**
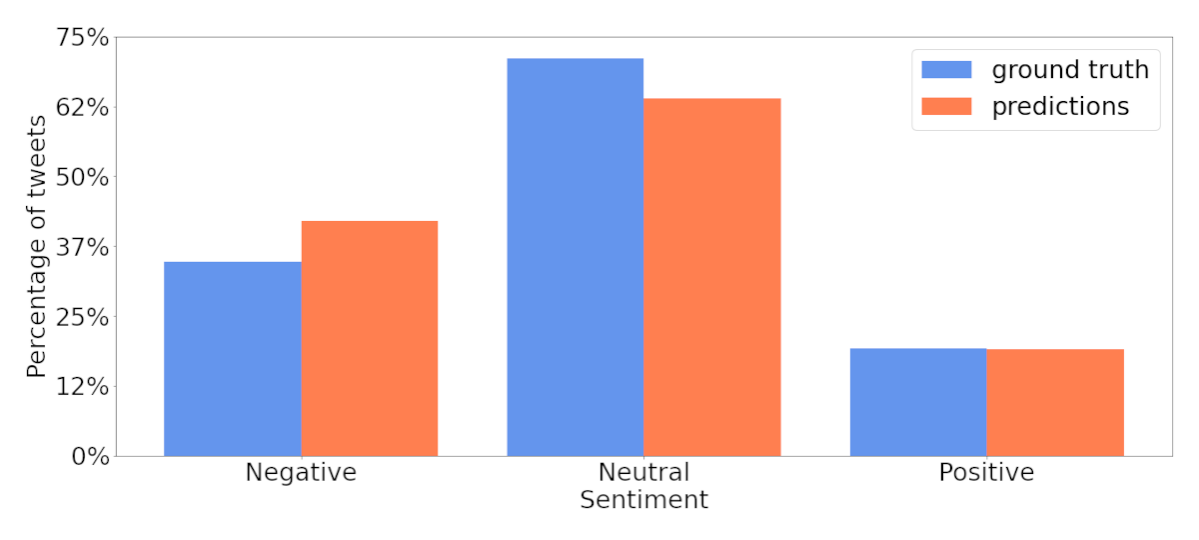
Comparison of the sentiment distributions of the manually annotated ground-truth labels (blue) and the model predictions (orange).

The performance of the Sentiment module on the real data was in line with that obtained on the benchmark data set, and its predictions were close to the ground truth. Figure 8 shows the sentiment distribution of the ground-truth labels (blue) and the predictions of the model (orange). The model leans slightly more toward negative sentiment. The performance (macroaveraged recall) on the subset of our data was 72.1. The model shows excellent generalization capabilities, which was in line with the performance recorded on the benchmark data set of 72.6 (SD 0.4).

To evaluate the Symptom Extraction module, we sampled our data set to have the same ratio of AE to no AE tweets as the benchmark data set SMM4H (57:43). The obtained relaxed F1 score was 63.3 (SD 0.7) (average over 10 sampling procedures), against 70.2 recorded on SMM4H. This gap in performance may be caused by the difference in the types of AEs present in the two data sets. For example, the benchmark data set focuses on sleep disorders and weight gain/loss, whereas the data we collected contain more instances of arm soreness and blood clotting, which the model had never encountered during training.

### Case Study: AstraZeneca

#### Overview

To demonstrate the possible uses of our monitoring system as a research tool, we created a brief report regarding the AstraZeneca vaccine. In particular, we focused on analyzing the phenomenon of the alleged correlation between the vaccine and some specific side effects (eg, blood clots), in comparison with the other monitored vaccines.

#### Sentiment Trends for AstraZeneca

**Figure 9:**
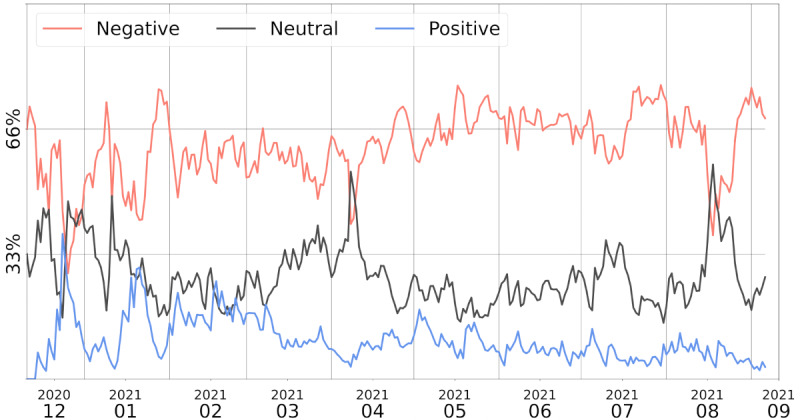
Monthly sentiment distribution in AstraZeneca vaccine–related tweets. The y-axis represents the percentage of negative (top, orange), neutral (middle, grey), and positive (bottom, blue) sentiment in the analyzed tweets. It is clear that the prevalent sentiment overall is “negative,” but we can observe spikes of nonnegativity in December and January.

We start by providing a general overview of the sentiment of the crowd toward the vaccine, and how it varied in time. Figure 9 shows the day-by-day percentage of positive, neutral, and negative tweets about the AstraZeneca vaccine from the day the monitoring started (December 11, 2020) to the most recent date at the time of writing (early September 2021).

We can see that the sentiment toward the vaccine has been mostly negative for the entire time period. This is likely due to the tendency of negative and worrying topics or critical opinions to spread more easily on the internet. Approximately one third of the tweets were neutral, corresponding to people sharing factual information about the vaccine or showing neutrality and detachment toward the topic.

There was a noticeable trend of “nonnegativity” between December and January, when positive and neutral tweets covered more than half of the discussion.

This might be related to the publication of an important study [48] about the efficacy of the AstraZeneca vaccine and its approval by the EMA.

#### Mentions of Thromboembolic Events

We then compared the frequency with which Twitter users mentioned AEs related to “thrombosis” and “blood clotting” compared to other vaccine side effects.

**Figure 10:**
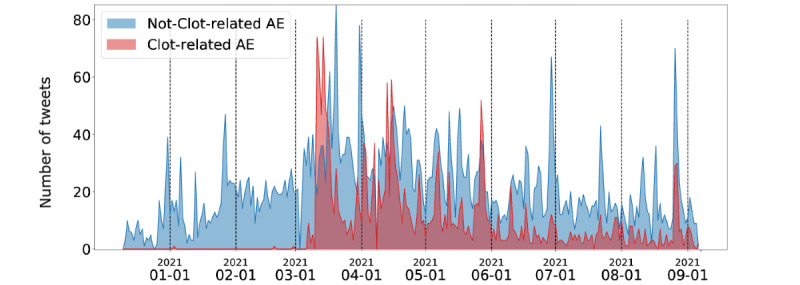
Number of tweets mentioning clot-related and nonclot-related keywords for the AstraZeneca vaccine (time is plotted on the x-axis and the number of tweets is plotted on the y-axis). The number of tweets mentioning clot-related adverse events (AEs) was initially next to zero, spiked in March 2021 due to media coverage, but has been gradually diminishing ever since. Tweets mentioning nonclot-related AEs show a more stable trend over time.

Figure 10 shows the number of detected tweets for each day that contained clot-related AEs (red series) and any other AE (blue series).

The absolute number of tweets discussing AstraZeneca and its AEs increased from December 2020 to February 2021; however, blood clotting events were rarely discussed on Twitter.

This changed in the first half of March 2021, when the number of tweets discussing clot-related AEs had a peak. At that time, some European states (eg, Germany) stopped inoculations of the AstraZeneca vaccine due to the possible correlation between the clots and the vaccine, along with some suspicious deaths from ischemia.

Since then, the public attention on clot-related AEs has remained high and peaked periodically (see the red series), without losing track of the other topics (the number of tweets discussing other AEs remained high).

As specified above, not all tweets with clot-related references are AE reports: most of them come from people sharing or commenting news pieces about the vaccine.

We can also observe that in the last month, the chatter about AstraZeneca has diminished, as the blue and red series report less than 20 tweets per day.

**Figure 11:**
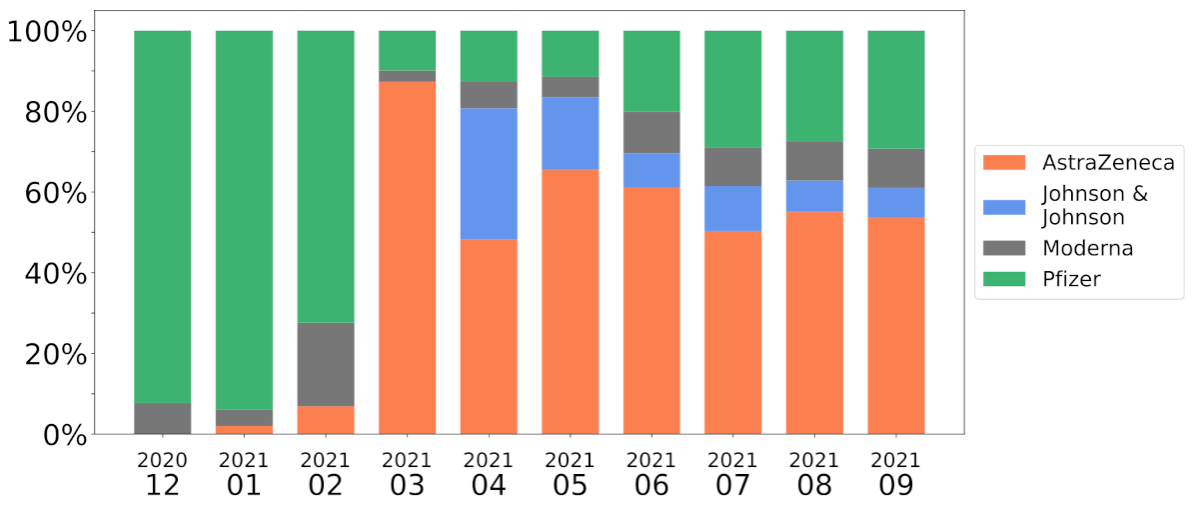
Monthly distribution of vaccine names mentioned in tweets with clot-related keywords (time on the x-axis, percentage of tweets on the y-axis). Most of the tweets were discussing clot-related adverse events connected to the Pfizer vaccine before March 2021, when the focus suddenly shifted to AstraZeneca.

Figure 11 offers a different perspective on the phenomenon: we collected all tweets mentioning blood clots and thrombosis, and divided them according to which vaccines they deal with. Before March 2021, most of the tweets dealing with clot-related AEs were associated with the Pfizer vaccine (75%-85%). With the wide news coverage about the cases related to AstraZeneca, the trend changed drastically, and over 80% of the tweets mentioning this kind of event were discussing AstraZeneca.

#### Evolution of Mentioned Symptoms Over Time

**Figure 12:**
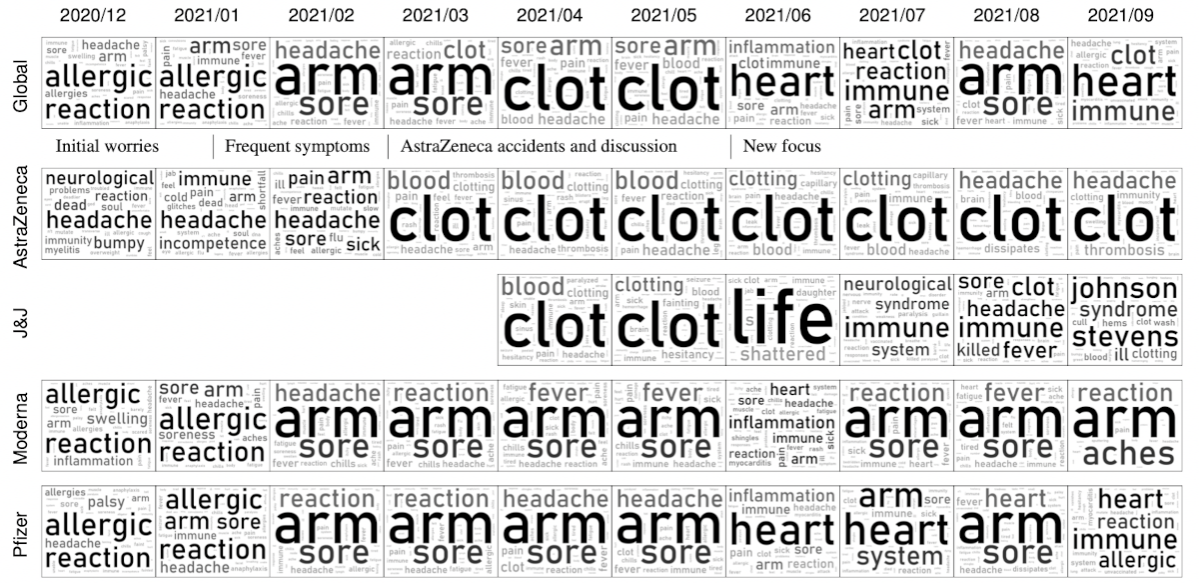
Evolution of the global word cloud (top row, all vaccines included) and the specific word clouds of the following vaccines: AstraZeneca, Johnson & Johnson (J&J), Moderna, Pfizer. The suspected adverse events were extracted using our model.

The wide news coverage had a strong influence on the topics of discussion among Twitter users. This can be seen even more clearly in Figure 12, which shows three series of word clouds that represent how the main topics discussed on Twitter varied in time. The first row shows the most frequent AEs globally discussed (considering all tweets) for each month. The following rows show the evolution of the topics for the tweets that mention AstraZeneca, Moderna, or Pfizer only.

In the first 2 months (December 2020 to January 2021), all of the discussions were focused on widespread worries and doubts of the users (eg, allergies, neurological problems, immune responses).

During the following months, as the vaccination campaign proceeded, the focus slowly shifted toward the most common side effects that the vaccinated population was experiencing (eg, soreness at the arm, feeling sick, headache).

The news about AstraZeneca in March caused a dramatic shift of topic, not only in the tweets regarding that particular vaccine but also globally: the word “clot” suddenly appears in the global word cloud and becomes the most discussed topic for the following months (this also influences Pfizer’s word cloud, where the “clot” topic becomes slightly visible in April).

**Figure 13:**
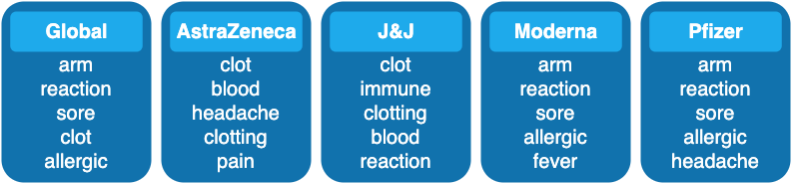
Top-5 most frequently mentioned terms globally and for the following vaccines: AstraZeneca, Johnson & Johnson (J&J), Moderna, Pfizer. This takes into consideration all of the collected tweets from December 15, 2020, to the beginning of September 2021.

Looking at the latest available data, we can see that “blood clots” are still the most trending topic for AstraZeneca, but the global discussion has finally moved toward other topics such as “heart” problems. That said, if we look at all of the collected data, from December 2020 to September 2021, “clot” is the fourth most mentioned term globally (Figure 13), surpassed in popularity only by the broader concepts “arm,” “reaction,” and “sore.” This shows how great of an impact this episode had on social media.

## Discussion

### Intended Use Cases

Our web portal could be useful for different categories of users.

The first category is the general public. Owing to the intuitive interface and graphics, generic users can keep themselves up to date and be made aware of the kind of news that is circulating, what symptoms are being discussed for the various vaccines, and under which terms.

The second category is journalists and news outlets. The section of the web portal dedicated to news trends might provide insights for the press to better understand the digital audience and help in fighting misinformation. The other information might be interesting to explore to discover the latest most discussed topics.

The third category concerns users in the health care sector. The information on the most shared symptoms and possible AEs might be helpful to point the attention of the experts toward particular effects of the new vaccines.

Finally, scholars working in the field of biomedical natural language processing can benefit from the portal. The code of the AE extraction architecture is publicly available, and the web portal includes an explanatory page about the various implemented modules. The objective is to raise interest of the natural language processing community on this topic, and open the door to suggestions and possible collaborations.

### Limitations

This project collects data from user-generated, unfiltered content, and makes use of automatic tools that have low and no human supervision. Therefore, it is important to highlight some limiting factors

The first limitation is the language barrier. As stated in the first sections, the current system is only able to analyze texts written in English. The COVID-19 vaccines are being distributed and discussed in several non-English–speaking countries, and therefore this data set is only a partial representation of public opinion. As stated in the *Data Collection* section, we plan to overcome this limitation with the use of multilingual models and/or automated translation services. We are already collecting tweets in other languages for the same time period, which will allow us to perform a complete comparative analysis in the future.

The second limitation relates to the demographics of Twitter users. Twitter is often used as a means to understand and monitor crowd opinions and real-world phenomena. However, it is not always the case that Twitter users are a representative sample of the population of interest. A population can be examined along various axes (eg, age, geography, gender, ethnicity), and specific social media environments tend to overrepresent some sets of the population (eg, users coming from densely populated areas, higher level of education, higher income or computer literacy) [49,50].

Bias and misinformation spread on social media. Social media are also infamous for the creation of echo chambers [51], where users of the same mindset end up aggregating. This can “artificially” increase engagement with polarizing posts, which in turn become more visible and gain more weight in the analyses. Social media are highly polarizing environments, in which shocking, controversial, and generally “negative” posts are rewarded (and therefore can be found more frequently in the collected data) [52,53]. Our system tries to cope with this by handling data deduplication (removing viral copy-pasted tweets) and collecting the most recent tweets (as opposed to the most popular). This, however, does not remove the threats of echo chambers and misinformation. As future work, we plan to add a new module based on our previous work [54] to better analyze phenomena related to the spread of misinformation.

Finally, the correctness of deep-learning modules remains an inherent limitation. Both the Sentiment Analysis and Symptom Extraction modules are machine-learning modules, and as such can perform prediction errors with a known probability. If the data are shown to the public, users must be aware that they have to be taken with a grain of salt. This is why, on our dashboard, we make sure to include a disclaimer to warn the user about this issue whenever we display data produced by machine-learning algorithms.

### Conclusions

We presented a tool connected with a web portal to monitor and display some key aspects of the public’s reaction to COVID-19 vaccines.

The idea was born from the awareness that, in the current phase of the pandemic, it is of key importance to create tools to monitor reactions, opinions, doubts, and feedback of the population on the vaccines. Social media are a precious source of raw information, which can be exploited to gain insights for pharmacovigilance purposes (guiding the attention of health care experts on emerging effects) and help in fighting misinformation.

The system also provides an overview of the opinions of the Twittersphere through graphic representations to make them accessible to different categories of users.

One of the main features of this tool is the extraction of suspected AEs from tweets with a deep-learning model, which proved to be reactive to the shifts of topic in the internet chatter. A future improvement could be the extraction of AEs from tweets of different languages, using a multilingual model or an automated translation service.

All code, tweet IDs, and the precomputed statistics of the collected tweets are available at GitHub [18].

## Abbreviations

AE: adverse event
API: application programming interface
BERT: bidirectional encoder representations from transformers
CRF: conditional random field
EMA: European Medicines Agency
MBFC: Media Bias/Fact Check
SMM4H: Social Media Mining for Health Applications
